# Ultrahigh‐Performance BiI_3_/CdSe Heterojunction Photodetectors With Broadband Response

**DOI:** 10.1002/nap2.70047

**Published:** 2026-03-20

**Authors:** Yifei Huang, Haiqiang Huang, Lujun Yang, Lei Liu, Hui Zhang, Jie Zheng, Yingkai Liu

**Affiliations:** ^1^ Yunnan Key Laboratory of Opto‐Electronic Information Technology Yunnan Normal University Kunming China; ^2^ Institute of Physics and Electronic Information Yunnan Normal University Kunming China; ^3^ Southwest United Graduate School Kunming China; ^4^ Key Laboratory of Advanced Technique & Preparation for Renewable Energy Materials Ministry of Education Yunnan Normal University Kunming China; ^5^ School Hospital of Yunnan Normal University Kunming China

**Keywords:** bismuth triiodide, broadband photodetection, heterojunction photodetectors, high responsivity, ultrafast response

## Abstract

Bismuth triiodide (BiI_3_) is a promising 2D metal halide for gamma/X‐ray detection, photovoltaics, and hybrid devices. In this work, two‐dimensional BiI_3_ nanosheets grown on vertical substrates were successfully prepared by physical vapor deposition. After that, a BiI_3_/CdSe heterojunction photodetector was constructed by a dry transfer process. It not only realized a broadband response in the visible range of 400–800 nm but also has excellent performance, including responsivity of up to 4.95 × 10^3^ A/W, a switching ratio of 0.97 × 10^6^, a response speed of 140/200 μs (rise/decay time), an external quantum efficiency of 1.45 × 10^6^%, and a specific detectivity of 1.25 × 10^14^ Jones. The photodetection mechanism was explored by photocurrent scanning imaging and energy band analysis. Imaging measurements confirmed its imaging capability with high resolution and high contrast. This study demonstrates the potential application of BiI_3_ materials in optoelectronic devices and provides new ideas for the development of a new generation of high‐performance photodetectors.

## Graphical abstract

A BiI_3_/CdSe van der Waals heterojunction photodetector is presented, integrating favorable material properties and optimized device architecture. The heterostructure facilitates efficient light absorption, interfacial charge transfer, and pronounced photoconductive gain, resulting in ultrahigh responsivity, detectivity, and high‐quality imaging performance, thereby demonstrating strong potential for next‐generation broadband optoelectronic and imaging applications.

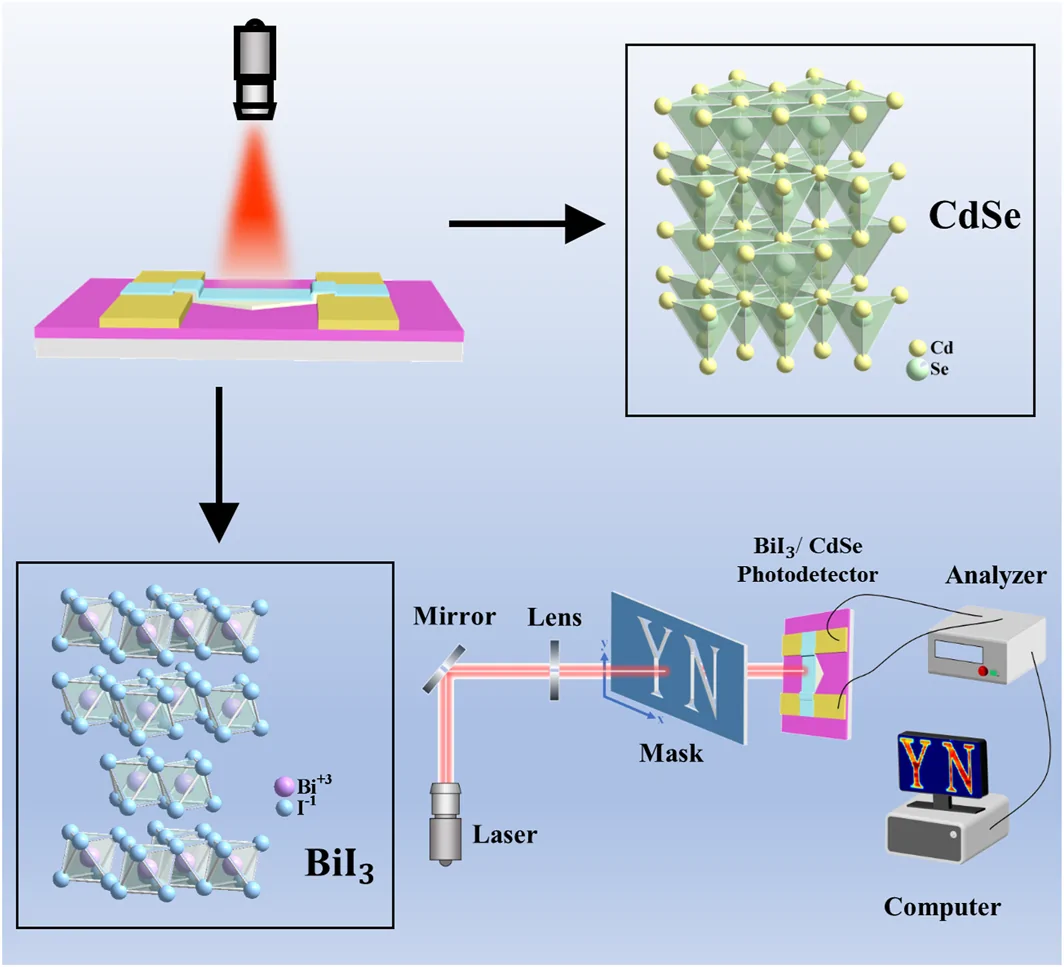

## 1 Introduction

Photodetectors (PDs) convert optical signals into electrical signals for information transmission and are indispensable devices in the modern information society [1, 2]. In recent years, new photoelectric materials have been widely used in optical communications, security monitoring, medical diagnosis, and other military and civilian fields [3, 4, 5, 6, 7]. Among them, two‐dimensional layered materials (2DLMs) have attracted much attention because of their novel physical effects [8, 9, 10]. PDs based on 2DLMs such as graphene, transition metal disulfides (e.g., MoS_2_ and WS_2_), metal‐sulfur compounds (e.g., InS_2_ and InSe), and metal halides (e.g., PbI_2_ and BiI_3_) have demonstrated excellent detection over a wide spectral range from UV to terahertz [11, 12, 13, 14, 15].

In particular, BiI_3_, as a wide‐bandgap (1.6–3.1 eV) semiconductor, combines high density and low toxicity, which makes it an ideal material for room‐temperature radiation/X‐ray detection [16, 17, 18, 19, 20], and exhibits excellent broad‐spectrum absorption properties in visible photovoltaics. Meanwhile, theories and experiments have shown that BiI_3_'s energy band structure is suitable for photovoltaic absorption, photoluminescence, solar cells, photoelectric signal converters, and sensors [21, 22, 23, 24, 25, 26, 27, 28, 29]. Zhou et al. pointed out that the weak interlayer coupling of monolayer BiI_3_ is suitable for large‐area film preparation [30]. Zhang et al. reported that the monolayer BiI_3_ has excellent thermal stability and visible light absorption from room temperature up to 600 K and that the conduction band position is suitable for the water redox reaction with an electron mobility as high as 200 cm^2^/Vs, indicating potential for photodetection and photocatalysis applications [31]. As one of the most promising materials in the new generation of photonics and optoelectronics, the 2D BiI_3_ material has triggered an extensive research boom.

Cadmium selenide (CdSe) is a II–VI semiconductor with a direct bandgap (1.7–2.4 eV), allowing it to provide excellent sensing at visible wavelengths by adsorbing relatively high incident photon energies [32, 33]. In addition, CdSe has excellent carrier transport properties and outstanding photovoltaic conversion efficiency [34, 35]. These advantages make CdSe an ideal candidate for hybrid structures. On the other hand, BiI_3_ features low dark current and strong optical absorption. Given their complementary properties, integrating BiI_3_ and CdSe into a heterojunction can promote efficient charge separation and broadband photodetection. Moreover, due to the significant difference in work functions between BiI_3_ and CdSe, their combination forms a unique and optimized Type‐II band structure. This configuration facilitates efficient separation of photogenerated carriers and generates strong photoconductive gain, thereby significantly promoting photodetection performance. Therefore, we consciously designed a hybrid structure of two‐dimensional BiI_3_ and CdSe and expect that this structure can generate excitation properties and improve its overall photoelectric performance.

In this study, vertically oriented BiI_3_ nanosheets were fabricated by a physical vapor deposition (PVD) method. This unique growth morphology facilitates subsequent transfer and integration with other materials. CdSe nanoribbons were then synthesized via chemical vapor deposition (CVD). The BiI_3_/CdSe heterojunction PD was then fabricated by a dry transfer technique. Compared to the single BiI_3_ device, the heterojunction device demonstrated significantly enhanced performance: The spectral response range was extended across the entire visible light spectrum (400–800 nm), with key parameters including ultrahigh responsivity (4.95 × 10^3^ A/W), large on/off ratio (0.97 × 10^6^), fast response time (140/200 μs), high external quantum efficiency (1.45 × 10^6^%), and ultrahigh specific detectivity (1.25 × 10^14^ Jones). Systematic photocurrent scanning imaging and band structure characterization revealed the photoelectric conversion mechanism of the heterojunction device, providing a theoretical explanation for the enhancement in performance of the device. Imaging tests further confirmed that it exhibited excellent performance with high resolution and contrast. This work offers an innovative solution for the fabrication of high‐performance photodetection devices and will contribute to their future commercial applications.

## 2 Experimental Section

### 2.1 Growth of BiI_3_ Nanosheets

BiI_3_ nanosheets were synthesized by PVD, and the synthesis setup is schematically illustrated in Supporting Information S1: Figure S1. BiI_3_ powder used as the source material was placed in the hot zone of the quartz tube, whereas the SiO_2_/Si substrate was positioned downstream from the source toward the cold end. The tube furnace was first preheated from room temperature to 150°C at a rate of 10°C/min. This was followed by a rapid temperature increase at a rate of 15°C/min to 340°C. The substrate and product were removed after holding at 340°C for 5 min and then cooling naturally. A constant argon flow of 6 sccm (standard state cubic centimeters/min) was used as a carrier throughout the synthesis.

### 2.2 Growth of CdSe Nanoribbons

CdSe nanoribbons were synthesized by the CVD method, and the synthesis setup is schematically shown in Supporting Information S1: Figure S2. CdSe powder as the source material was placed in the hot zone of a quartz tube, whereas clean SiO_2_/Si substrates sputtered with gold particles for 100 s were placed upstream and downstream of the ceramic boat. The tube furnace was then heated at a rate of 15°C/min from room temperature to 825°C. The tube furnace was then kept at 825°C for 140 min and then cooled naturally before the substrate and product were finally removed. A constant flow of argon (with 5% H_2_) at 15 sccm (standard state cubic centimeters/min) was used as a carrier throughout the synthesis.

### 2.3 Preparation of BiI_3_/CdSe Heterojunction Photodetector

The photodetector, composed of a BiI_3_/CdSe heterostructure and two electrodes, was fabricated by a dry transfer method. First, In/Au (10/50 nm) electrodes were deposited onto a 285 nm SiO_2_‐Si substrate using photolithography and thermal evaporation. Next, BiI_3_ and CdSe materials were transferred from a silicon wafer to a PDMS (polydimethylsiloxane) substrate on a 2D transfer platform. The sample on PDMS was then transferred to the Au electrodes on the SiO_2_‐Si substrate to fabricate the device. Finally, the device was annealed for 30 min at 80°C in an Ar atmosphere to remove impurities and achieve good contact. The fabrication process is also shown in Supporting Information S1: Figure S3.

### 2.4 Characterization

BiI_3_ and CdSe samples were characterized using optical microscopy, energy‐dispersive X‐ray spectroscopy (EDS, K‐Alpha+, USA), transmission electron microscopy (TEM, Talos P200X G2, America), X‐ray diffraction (XRD, DX 2700, China), atomic force microscopy (AFM, Bruker Dimension Icon, Germany), Raman spectroscopy (Andor, UK), PL spectroscopy and attenuation curve analysis (IK 3301, Japan), UV absorption spectrophotometry (ESCALAB Xi+, America), X‐ray photoelectron spectroscopy (XPS, Thermo Fisher Scientific ESCALAB, America), and UV photoelectron spectroscopy (UPS, ESCALAB Xi+, America). Subsequently, the optical and electrical properties of the BiI_3_ and BiI_3_/CdSe photodetectors were measured using a semiconductor integrated test system (Keithley 4200‐SCS, America).

## 3 Results and Discussion

The preparation of BiI_3_ nanosheets (NSs) is schematically depicted in Supporting Information S1: Figure S1. BiI_3_ powder sublimates in the high‐temperature region of the quartz tube, and the vapors are swept forward by a continuous flow of argon gas and then crystallize into vertically growing NSs and microparticles in the low‐temperature region, as shown by the red area in the upper left of Figure 1b and its inset. It was found that vertically grown BiI_3_ NSs have a smaller contact area compared to other horizontally grown 2D materials, which means that there is limited chemical bonding between vertically grown BiI_3_ NSs and SiO_2_/Si substrates, and this vertical product can be more easily transferred and composited with other materials. A SEM image of a single BiI_3_ NS is shown in the upper right of Figure 1b, revealing a distinctive semi‐hexagonal shape with clear and sharp edges. The smooth surface indicates that the sample has good crystallinity. An energy‐dispersive X‐ray spectroscopy (EDS) was also explored to examine the chemical composition and elemental distribution of the BiI_3_ NSs, which are displayed in the lower left and lower right panels of Figure 1b. It illustrates that the elements Bi and I are uniformly distributed throughout the BiI_3_ NSs. The EDS of BiI_3_ is presented in Figure 2a. It reveals that no other elements were detected except Bi and I. The atomic ratio of Bi and I is about 1:3, which is well compatible with the stoichiometric ratio of BiI_3_. The chemical composition and valence states of BiI_3_ NSs were investigated by X‐ray photoelectron spectroscopy (XPS) to collect Bi 4f and I 3d spectra, as shown in Supporting Information S1: Figure S4a–c. The peaks located at 159.2 and 164.5 eV match Bi 4f_7/2_ and Bi 4f_5/2_, respectively. Two strong peaks at 619.1 and 630.5 eV were attributed to I 3d_5/2_ and I 3d_3/2_, further confirming that the valence states of Bi and I are +3 and −1, respectively.

**FIGURE 1:**
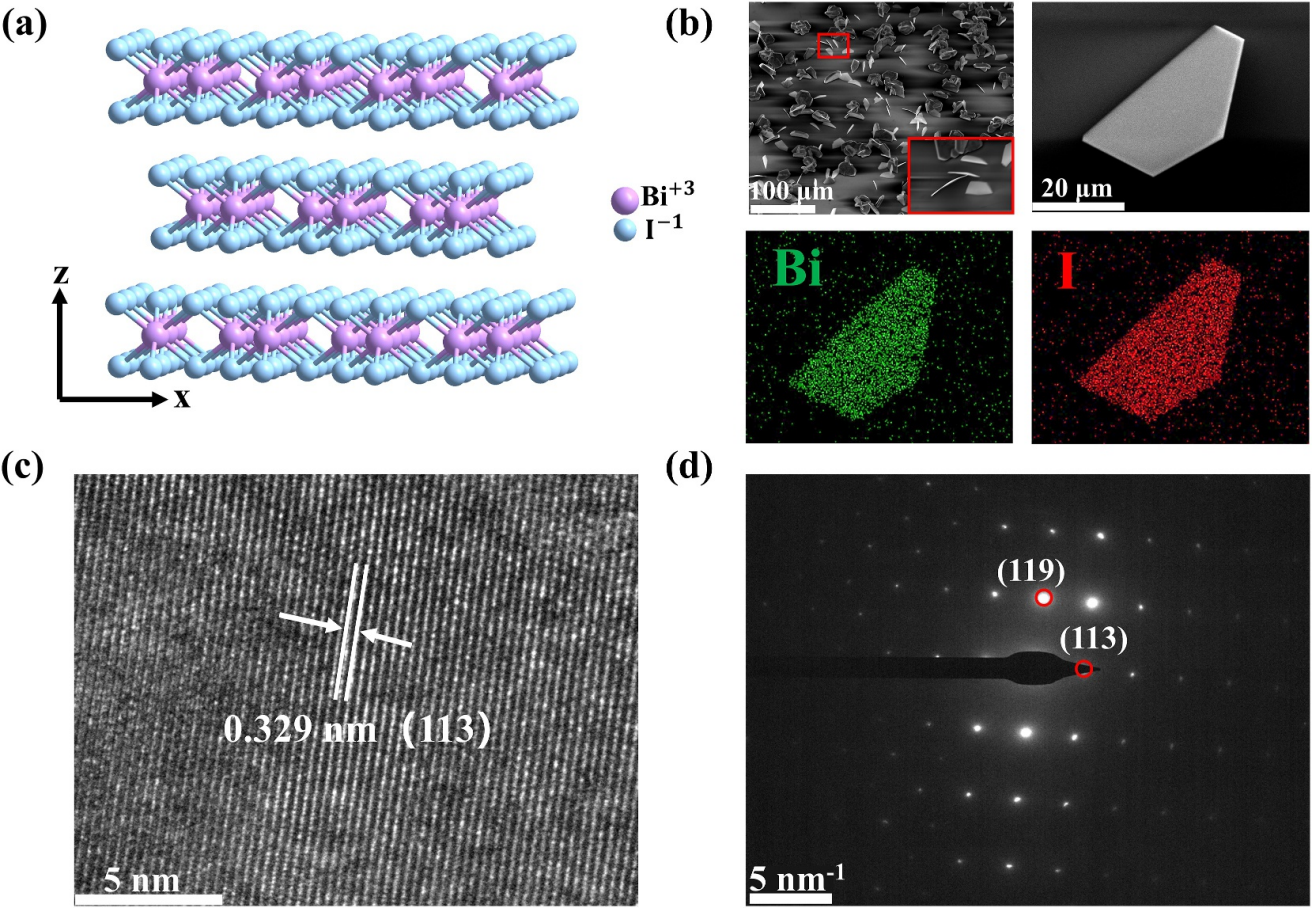
Microstructure characterization of BiI_3_ NSs. (a) Layered crystal structure of BiI_3_. The purple and blue spheres correspond to bismuth and iodine, respectively. The black vectors indicate the orthogonal axes in real space. (b) Scanning electron microscope images of BiI_3_ (red boxes show magnified SEM images of selected regions) and EDS elemental mapping of BiI_3_ NSs. (c) and (d) HRTEM and SAED images of the grown BiI_3_ NSs.

**FIGURE 2:**
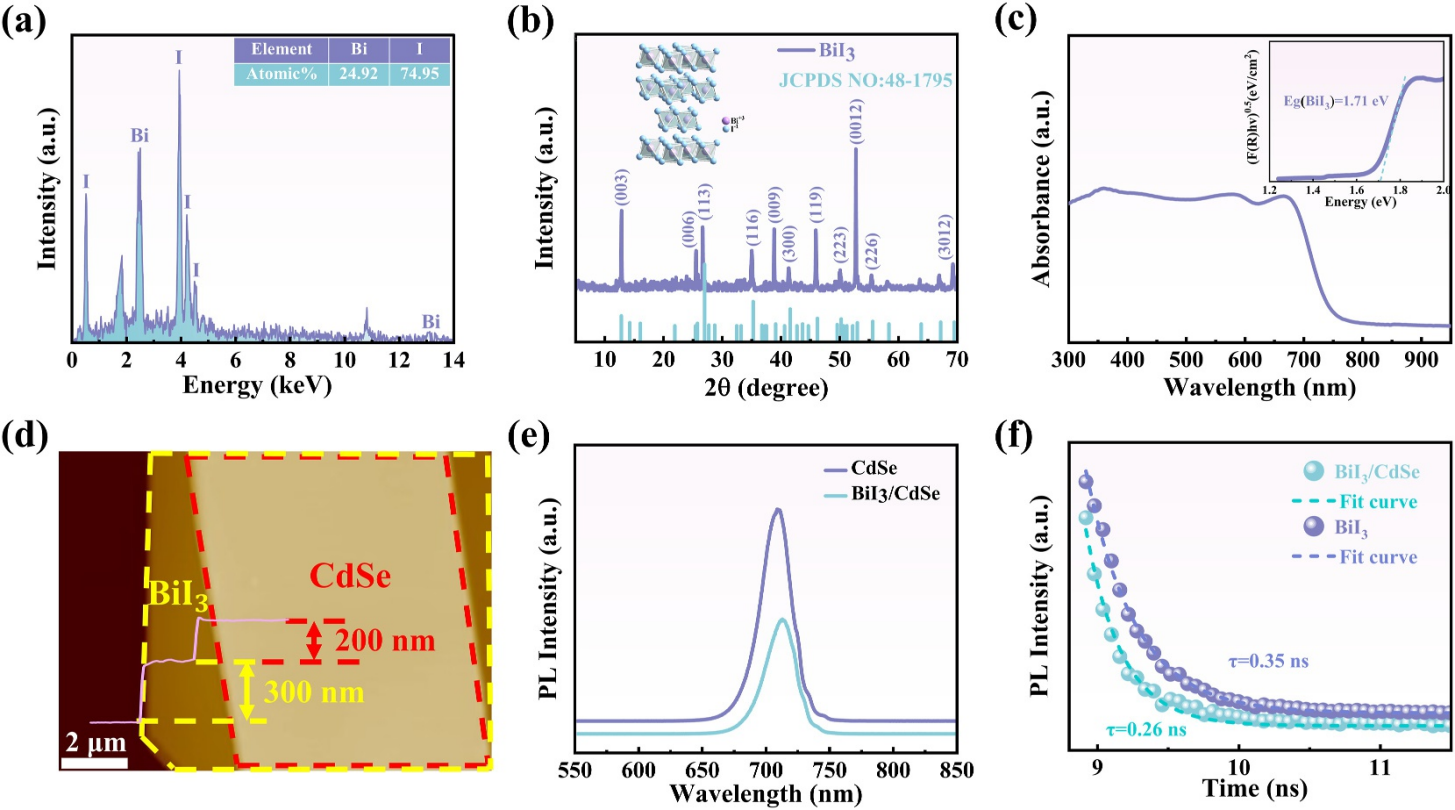
(a) EDS elemental composition of BiI_3_ NSs in the growing state. (b) XRD pattern of BiI_3_ NSs. (c) UV–Vis absorption spectra and Tauc plots of BiI_3_ nanosheets. (d) AFM image of the BiI_3_/CdSe heterojunction. (e) PL spectra of CdSe and the BiI_3_/CdSe heterojunction. (f) Time‐resolved PL spectra of pure BiI_3_ and the BiI_3_/CdSe heterojunction.

The crystal structure of the BiI_3_ NSs was characterized by transmission electron microscopy, and the obtained high‐resolution transmission electron microscopy (HRTEM) and selected area electron diffraction (SAED) are shown in Figure 1c,d. The SAED pattern confirms the single‐crystal quality and can be indexed to a hexagonal structure with lattice parameters *a* = 7.519 Å and *c* = 20.721 Å (PDF#48‐1795). The corresponding HRTEM image in Figure 1d further confirms the high‐quality single‐crystal nature of the NS. The lattice spacing between adjacent planes is 0.329 nm, which corresponds to the (113) crystal plane.

The phase identification of BiI_3_ NSs was followed by an X‐ray diffraction. Its XRD pattern is shown in Figure 2b. The main diffraction peaks located at 12.84°, 26.64°, 38.82°, 45.92°, and 52.74° were indexed to the (003), (113), (009), (119), and (0012) crystal planes, respectively, which match the hexagonal structure of BiI_3_ [36] (PDF#48‐1795). The sharp diffraction peaks indicate that these NSs are well crystallized.

Figure 2c and Supporting Information S1: Figure S4d show the UV–Vis absorption spectra of BiI_3_ NSs and CdSe nanoribbons, which are given by the Tauc diagram [37]:

(1) $$\begin{array}{c} {\left(\alpha h\nu \right)}^{\frac{1}{n}}=A\left(h\nu -E_{g}\right) \end{array}$$

where *α*, *h*, *ν*, *A*, and *E*_*g*_ are denoted as the absorption coefficient, Planck's constant, the light frequency, the proportional constant, and the bandgap, respectively. *n* equals 1/2 for direct bandgap materials and 2 for indirect bandgap materials. The optical bandgaps of BiI_3_ and CdSe were calculated to be 1.71 and 1.74 eV, respectively, as shown in the inset of Figure 2c and Supporting Information S1: Figure S4d, which are consistent with previous reports in the literature [38, 39]. Figure 2d shows the AFM image of the BiI_3_/CdSe heterojunction. It is seen that the thicknesses of the BiI_3_ NSs and CdSe nanoribbons are 300 and 200 nm, respectively. The fluorescence spectra of both the CdSe nanoribbons and the BiI_3_/CdSe heterojunction exhibit a fluorescence peak at 710 nm, which is consistent with the fluorescence characteristic peak of the single CdSe nanoribbons. It is noteworthy that the PL intensity of the BiI_3_/CdSe heterojunction exhibits significant fluorescence quenching in Figure 2e. This significant quenching of PL intensity may be attributed to carrier relaxation paths and charge transfer between CdSe and BiI_3_. Figure 2f represents the time‐resolved PL spectra of single BiI_3_ and BiI_3_/CdSe. The PL lifetime of single BiI_3_ is found to be ∼0.35 ns, whereas that of the BiI_3_/CdSe heterojunction is reduced to 0.26 ns. This reduction in the PL lifetime suggests that efficient charge carrier separation at the heterojunction interface is realized, which fully justifies the significant advantages of this structure for high‐performance photodetector applications [40, 41].

Figure 3a demonstrates the spectral response characteristics of the single‐component BiI_3_ device in the range of 400–800 nm, whose response peaks are at 510 and 635 nm with responsivities (*R*) of 1.20 and 1.67 A/W, respectively. In contrast, the BiI_3_/CdSe heterojunction device in the same spectral range elucidates significantly enhanced photoresponsive performance (Figure 3b). It is evidenced that the responsivities at 510, 635, and 685 nm are 5.32 × 10^3^, 5.95 × 10^3^, and 6.56 × 10^3^ A/W, respectively, which is an enhancement of three orders of magnitude over the single‐component device. Notably, the heterojunction device not only realizes an order‐of‐magnitude increase in responsivity but also significantly broadens its effective response range and maintains excellent performance throughout the visible range of 400–800 nm. Figure 3c,d highlights the current‐voltage (I‐V) characteristic curves of a single BiI_3_ device and a BiI_3_/CdSe heterojunction in the dark and under 635 nm illumination (0.24 mW/cm^2^), respectively. It was realized that the photo/dark current of a single BiI_3_ device is 3.02 × 10^−10^/3.62 × 10^−13^ A with an on/off ratio of 8.3 × 10^2^ (Figure 3c), and the on/off ratio for a single CdSe device is 3.52 × 10^3^ (Supporting Information S1: Figure S5a). In comparison, for the BiI_3_/CdSe heterojunction device, it yields significantly enhanced figures of merit: The photocurrent is abruptly increased by three orders of magnitude to 8.03 × 10^−7^ A, whereas the dark current is only slightly increased to 5.53 × 10^−13^ A. To verify its reliability and reproducibility, three independent devices were tested under identical conditions (Supporting Information S1: Figure S6), and the dark current values of them are on the order of 10^−13^ A. Thus, the heterojunction device generated an outstanding current on/off ratio of 1.45 × 10^6^ and a 1.75 × 10^3^‐fold increase over a single device (Figure 3d).

**FIGURE 3:**
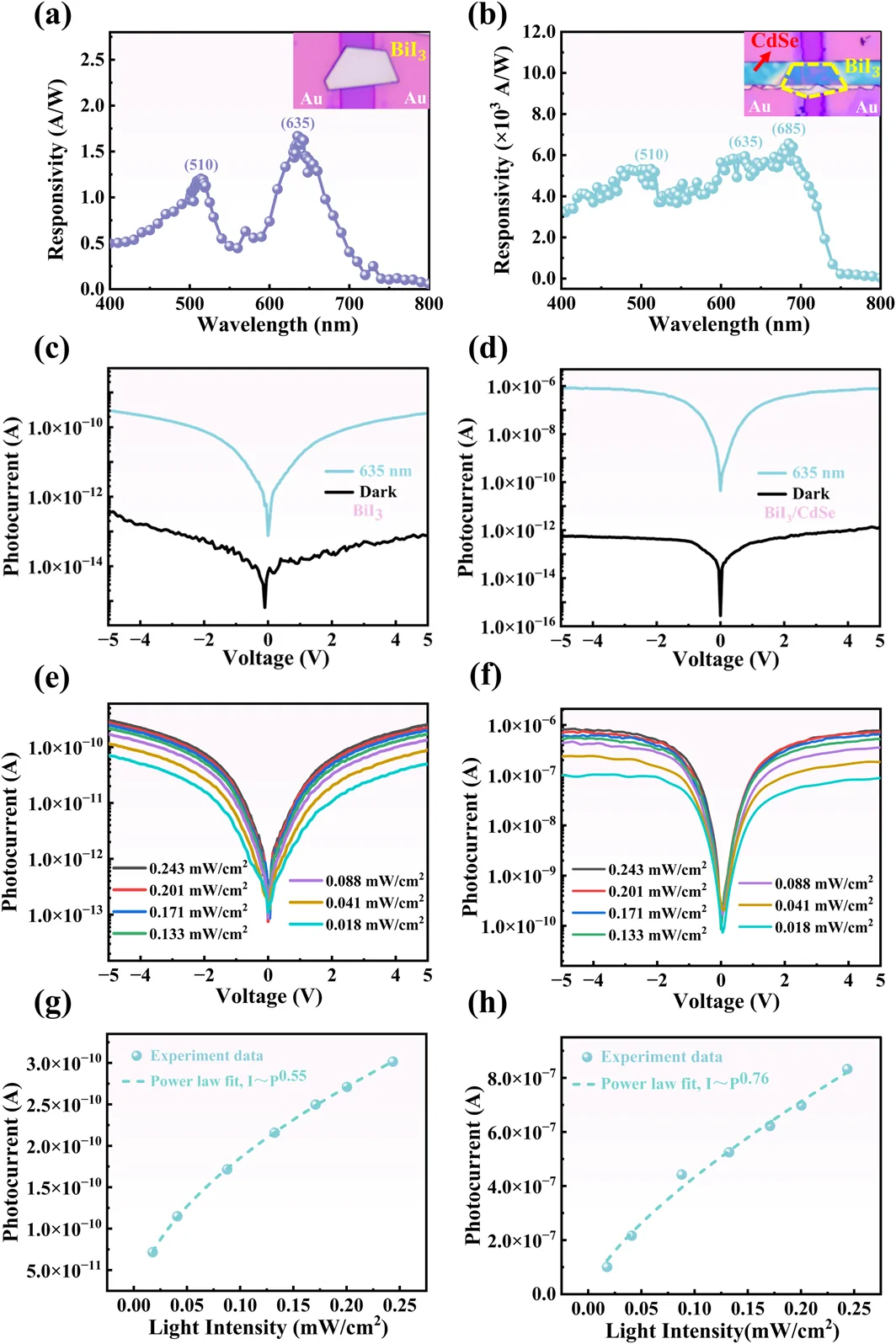
Spectral responsivity (R) of BiI_3_ and BiI_3_/CdSe photodetectors under 5 V bias and their optoelectronic properties under 635 nm light. (a) Response spectrum of the BiI_3_ photodetector; inset: an optical microscope image of the device. (b) Response spectrum of the BiI_3_/CdSe photodetector; inset: an optical microscope image of the device. (c) Log I‐V characteristic curves of BiI_3_. (d) BiI_3_/CdSe photodetectors in the dark and under 635 nm light, respectively. (e) Log I‐V characteristic curves of BiI_3_. (f) BiI_3_/CdSe photodetectors at different optical power densities, respectively. Experimental and fitted relationships between photocurrent and optical intensity for (g) BiI_3_ and (h) BiI_3_/CdSe photodetectors.

In addition, we further compare the photocurrent (*I*_ph_) versus incident optical power density (P) for the two devices, as portrayed in Figure 3e,f. We fitted them using the power‐law equation *I*_ph_ = AP^θ^ [42], where *I*_ph_ is the photocurrent, A is the scaling factor, P is the optical power density, and the exponent *θ* is the fitting coefficient. Notably, the photocurrent also gives rise to an increasing trend with increasing power density, implying that the carrier generation efficiency is positively correlated with the absorbed photon flux [43]. *θ* reflects the pairing, trapping, and recombination of photogenerated electron–hole pairs. The corresponding *θ* values for BiI_3_ and BiI_3_/CdSe composite devices were found to be 0.55 and 0.76, respectively, as visualized in Figure 3g,h. The nearly linear relationship between photocurrent and optical power density highlights the excellent photon‐electron conversion efficiency of the prepared devices [44].

The key metrics of photodetectors include spectral responsivity (*R*) [45], external quantum efficiency (EQE) [46], and specific detectivity (D*) [47]. In order to systematically evaluate the performance advantages of BiI_3_/CdSe heterojunction devices over single‐component BiI_3_ devices, quantitative calculations and comparative analyses of the above parameters were carried out using the following equations:

(2) $$\begin{array}{c} R=\frac{I_{ph}-I_{d}}{PA} \end{array}$$

(3) $$\begin{array}{c} \text{EQE}=R\frac{hc}{q\lambda } \end{array}$$

(4) $$\begin{array}{c} D^{*}=R\sqrt{\frac{A}{2eI_{d}}} \end{array}$$

where *I*_*ph*_, *I*_*d*_, *P*, and *A* denote the photocurrent, the dark current, the incident optical power density, and the effective illumination area, respectively. *q* is the elementary charge, *h* is Planck's constant, *c* is the speed of light, and *λ* is the incident light wavelength.

Figure 4a–d systematically compares the variation patterns of *R*, D*, and EQE with power density for single BiI3 devices and BiI3/CdSe heterojunction devices under 635 nm light illumination with power density from 18 to 243 μW/cm^2^ at a bias of 5 V. It confirms that the photogenerated carrier complex at the interface is enhanced with increasing optical power density, whereas *R*, D*, and EQE yield the reverse trend [48]. For the single BiI_3_ device, the best figures of merit (*R*, EQE, and D*) at 635 nm with a power density of 18 μW/cm^2^ are 3.77 A/W, 918.7%, and 1.19 × 10^11^ Jones, whereas those of the BiI_3_/CdSe composite device are 4.95 × 10^3^ A/W, 0.97 × 10^6^%, and 1.25 × 10^14^ Jones, which were improved by three orders of magnitude. It should be noted that the D* calculated from Equation (4) may be overestimated because we only considered the shot noise of the dark current. To address this problem, we further measured the noise power spectra of the two devices (Figure 4e). In the low‐frequency region, the flicker (1/f) noise dominates the total noise. Therefore, we further analyzed the effect of low‐frequency noise current on D* and recalculated D* using the flicker noise according to the following formula [49]:

(5) $$\begin{array}{c} D^{*}=\frac{\sqrt{AB}}{\text{NEP}}=\frac{R\sqrt{AB}}{I_{\text{noise}}}=\frac{R\sqrt{A}}{S_{n}} \end{array}$$

where *A* is the active area, *B* is the electrical bandwidth, and *S*_*n*_ is the noise spectral density. Figure 4f shows the frequency‐dependent D* of the BiI_3_ photodetector and the BiI_3_/CdSe heterojunction photodetector. It can be seen that the BiI_3_/CdSe device exhibits excellent performance, maintaining a detectivity as high as 3.60 × 10^13^ Jones even at a low frequency of 1 Hz. This value is significantly higher than that of typical commercial Si‐based photodetectors (≈ 10^12^ Jones) [50]. Based on the fact that the single device and hybrid device have a broadband response in the range of 400–800 nm, we further measured the optoelectronic properties of them at the wavelengths of 510 and 685 nm, which are depicted in Supporting Information S1: Figures S7 and S8. It reveals that the BiI_3_/CdSe device exhibits excellent optoelectronic performance under illumination at 510 and 685 nm. It is concluded that the three characteristic wavelengths (510/635/685 nm) result in all verifying the wideband responsiveness of the heterojunction device in the range of 400–800 nm. Moreover, it achieves a leapfrog enhancement of at least three orders of magnitude in *R*, EQE, and D* over the single‐component BiI_3_ device. The above‐mentioned enhancements mainly stem from its high photocurrent and low dark current characteristics. Meanwhile, compared with the single CdSe device, which exhibits a responsivity of 1.12 × 10^3^ A/W, an EQE of 2.19 × 10^5^%, and a detectivity of 4.25 × 10^12^ Jones (Supporting Information S1: Figure S5b–d), the BiI_3_/CdSe heterojunction device still demonstrates superior overall performance, further demonstrating the pronounced performance advantages of the BiI_3_/CdSe device.

**FIGURE 4:**
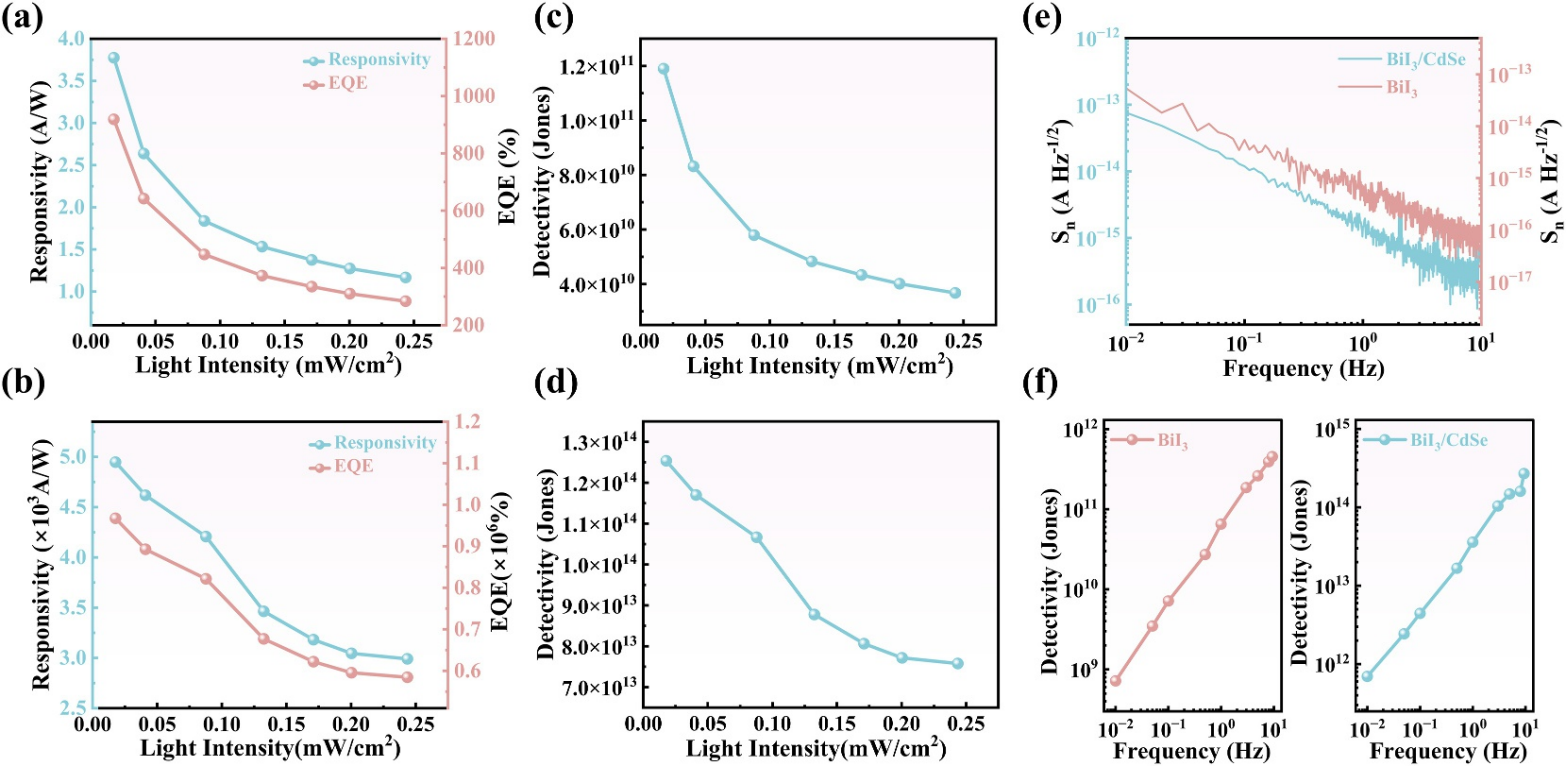
Responsivity, EQE, and detectivity of BiI_3_ and BiI_3_/CdSe photodetectors under 635 nm light illumination with power density from 18 to 243 μW/cm^2^ at a bias of 5 V. *R* and EQE values of (a) BiI_3_ and (b) BiI_3_/CdSe photodetectors at different optical power densities. (c) D* of BiI_3_ and (d) BiI_3_/CdSe photodetectors at different optical power densities. (e) Noise power spectra of BiI_3_ and BiI_3_/CdSe photodetectors under dark conditions. (f) D* at different frequencies of BiI_3_ and BiI_3_/CdSe photodetectors.

Temporal response is another parameter of photodetectors. To investigate the photoresponse dynamics of the two devices, we applied on/off light modulation by adding an optical chopper into the system and measured the rise/decay time. Figure 5a,c demonstrates the transient photoresponse characteristics of a single BiI_3_ device and a BiI_3_/CdSe heterojunction device to a pulsed signal at a frequency of 700 Hz, respectively. It is seen that the on/off switching behavior of the single and composite devices remains stable over multiple cycles. In a switching cycle, the rise time is defined as the time required for the photocurrent to increase from 10% to 90%, whereas the decay time is defined as the time required for the photocurrent to decrease from 90% to 10%. Thus, the rise/decay time of the single BiI_3_ device is 2.95/2.59 ms in Figure 5b, whereas that of the BiI_3_/CdSe composite device is 140/200 μs in Figure 5d. Compared with that of the single device, the response speed of the heterojunction device was significantly improved, indicating efficient separation of photogenerated electron–hole pairs at the interface.

**FIGURE 5:**
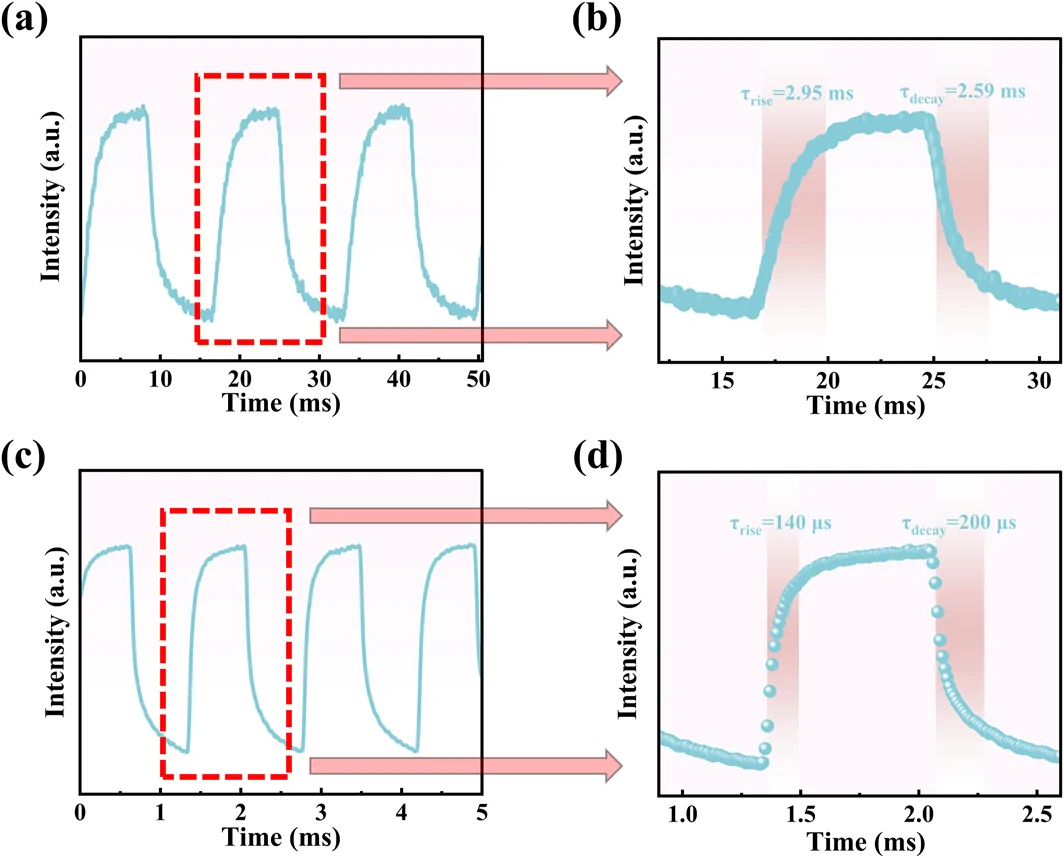
Photoresponse characteristics of BiI_3_ and BiI_3_/CdSe photodetectors. (a) Response curve of the BiI_3_ photodetector at 635 nm. (b) Response velocity of the BiI_3_ photodetector at 635 nm. (c) Response curve of the BiI_3_/CdSe photodetector at 635 nm. (d) Response velocity of the BiI_3_/CdSe photodetector at 635 nm.

We further investigated the stability and the effect of prolonged illumination, as well as ambient conditions, on our device. The operational stability was evaluated under continuous illumination (1–60 min) and after 30 days of ambient storage (≈ 22°C, relative humidity ≈ 40%), As shown in Supporting Information S1: Figure S9, the photocurrent remained largely stable under continuous illumination, with only slight decreases due to carrier saturation, consistent with the power‐law dependence on light intensity (*I*_ph_ = AP^*θ*^, *θ* < 1; see Figure 3h). Both BiI_3_ and BiI_3_/CdSe devices retained over 80% of their initial photocurrent after 30 days, with nearly unchanged dark current and a high on/off ratio (∼10^5^), demonstrating excellent stability under prolonged illumination and ambient conditions.

In order to explore the photocurrent generation and transport mechanism of the heterojunction device more deeply, scanning photocurrent imaging analysis was performed at a laser wavelength of 638 nm and a bias voltage of 5 V. Figure 6b corresponds to the red translucent region in Figure 6a. The photocurrent response of the overlapped region of BiI_3_ and CdSe in the channel is significantly higher than that of other regions, suggesting that the heterojunction has an important role in inducing the generation and separation of photogenerated electron–hole pairs. In order to elucidate the photoconversion mechanism of BiI_3_/CdSe photodetectors, we propose a theoretical model of the carrier transport process based on the energy band structure. The energy band evolution of BiI_3_ before and after contact with CdSe material was measured by UV photoelectron spectroscopy (UPS), as displayed in Figure 6c,d. The cut‐off edges (*E*_cut‐off_) of BiI_3_ and CdSe nanoribbons were found to be 17.14 and 16.20 eV (Supporting Information S1: Figure S10), respectively. By subtracting *E*_cut‐off_ from the UV incident energy (21.22 eV) [51], the corresponding work functions of BiI_3_ NSs and CdSe nanoribbons are 4.08 and 5.02 eV, respectively. The valence band edge (*E*_*V*_) and the conduction band edge (*E*_*C*_) were obtained by the following equations:

(6) $$\begin{array}{c} E_{V}=h\nu -E_{\text{cutoff}}+\Delta E \end{array}$$

(7) $$\begin{array}{c} E_{c}=E_{v}-E_{g} \end{array}$$

**FIGURE 6:**
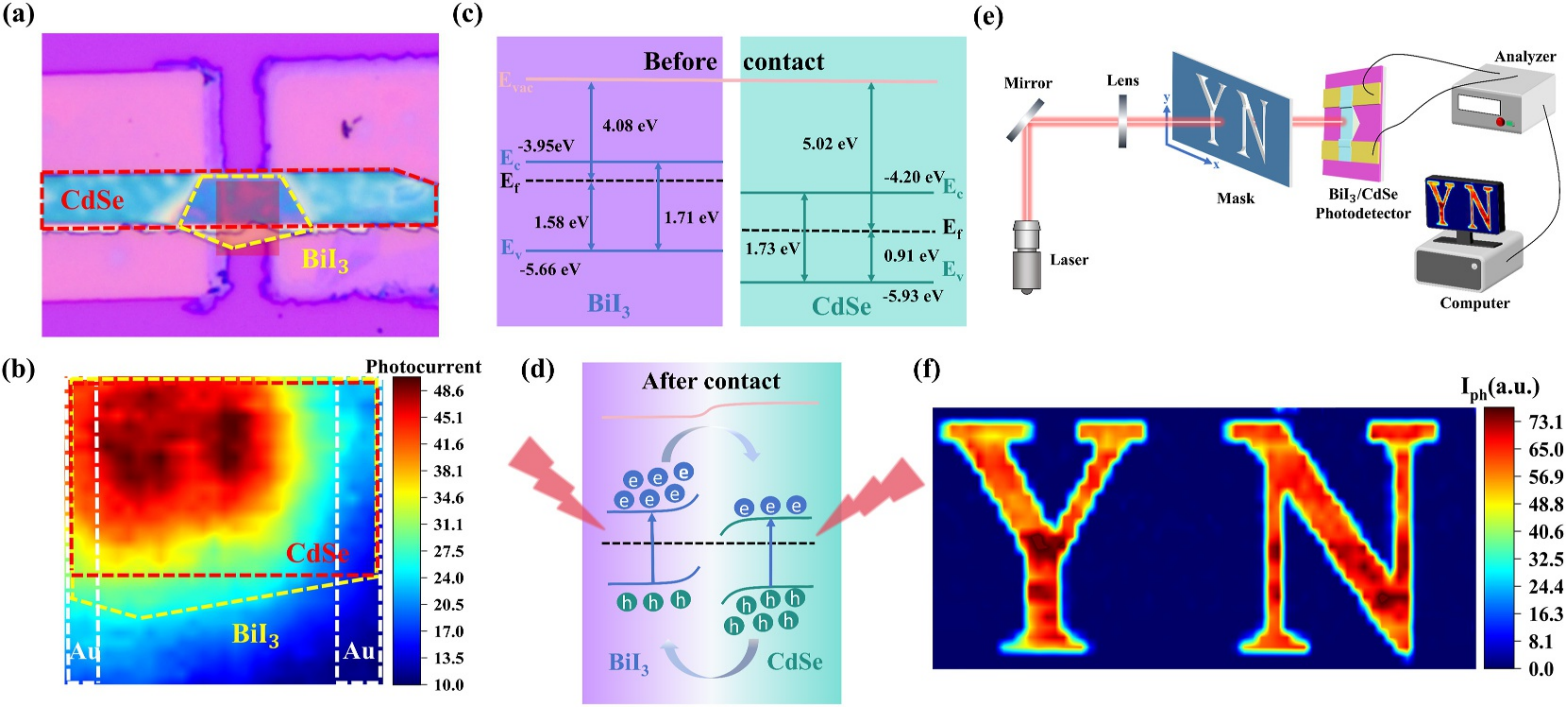
Scanning photocurrent mapping and energy band structure of the BiI_3_/CdSe device. (a) Optical image of the BiI_3_/CdSe device. (b) Scanning photocurrent mapping of the BiI_3_/CdSe device (V_ds_ = 5 V), with the red, yellow, and white dashed lines denoting the profiles of CdSe, BiI_3_, and the electrodes, respectively. (c, d) The contact between the BiI_3_ and CdSe energy band structures before and after contact. (e) Schematic of the image scanning system. (f) Imaging results corresponding to the “YN” pattern under 638 nm light illumination.

The *E*_*V*_ and *E*_*C*_ values were −5.66 and −3.95 eV for BiI_3_ and −5.93 and −4.20 eV for CdSe, respectively (Figure 6c). When the two materials are in contact, the energy band arrangement of the BiI_3_/CdSe heterojunction exhibits a typical Type‐II energy band interleaved structure (Figure 6d), which stems from the fact that both the conduction (−3.95 eV) and valence (−5.66 eV) band energy levels of BiI_3_ are higher than those of the CdSe (conduction band −4.20 eV, valence band −5.93 eV). In addition, an interfacial potential barrier of 0.94 eV is formed at the interface region due to the work function difference between BiI_3_ and CdSe. This barrier can effectively inhibit carrier transport under dark‐state conditions, inhibiting the dark current (5.53 × 10^−13^ A). The potential barrier can also repress photogenerated carriers in the interface region. On the other hand, a large number of photogenerated carriers are generated and accumulated at the interface under light irradiation, resulting in a decrease in the potential barrier [52]. Thus, the excitons in the two components can be efficiently separated through the overlap region; that is, electrons are injected into the CdSe conduction band, and holes are reversely injected into the BiI_3_ valence band, which is consistent with the previous photocurrent mapping results. Driven by the applied bias voltage, the photogenerated electrons (holes) are injected into the external circuit in a directional manner, which leads to significant photoconductive gain in the BiI_3_/CdSe heterojunction device [53]. This charge modulation mechanism based on energy band engineering has been proven to be an effective way to promote the performance of heterojunction devices.

Considering the remarkable photosensitizing properties of BiI_3_/CdSe photodetectors in the visible region, we further explored their imaging capabilities through an imaging system. An image scanning system is shown in Figure 6e, where the BiI_3_/CdSe photodetector is employed as a signal receiver. The positions of the photodetector and the laser are kept relatively fixed, and a mask plate with a skeletonized “YN” pattern is placed between them. The laser beam comes through the skeletonized pattern and reaches the surface of the photodetector. The movement of the mask plate in the *X* and *Y* directions can be precisely adjusted by a controller. When the laser scans the patterned mask, the semiconductor analyzer simultaneously measures and records the current signal and then converts it into a high‐resolution “YN” image, as displayed in Figure 6f. The “YN” image on the screen indicates that the BiI_3_/CdSe photodetector has excellent high‐resolution and high‐contrast imaging capability. To further quantitatively evaluate the imaging quality of the device, we calculated its signal‐to‐noise ratio (SNR), defined as follows [54]:

(8) $$\begin{array}{c} \text{SNR}=20\log\frac{I_{i}}{I_{0}} \end{array}$$

where *I*_*i*_ and *I*_0_ represent the measured current of the device with and without laser irradiation, respectively. As shown in Supporting Information S1: Figure S11, the SNR of the device under different optical powers is presented. The results indicate that at the maximum illumination, the device achieves an SNR as high as 123 dB, demonstrating its capability to achieve high‐quality imaging even under weak light conditions. The above results further confirm that the BiI_3_/CdSe heterojunction photodetector has significant potential in the fields of high‐resolution image archiving, optical communication technology, and medical imaging.

Figure 7a,b systematically compares the key performance parameters of our photodetector with the reported BiI3‐based and other iodide‐based photodetectors [15, 28, 55, 56, 57, 58, 59, 60, 61, 62], whose test conditions are depicted in Supporting Information S1: Figure S12. It is found that the BiI_3_/CdSe heterojunction photodetector achieves excellent comprehensive performance advantages. It not only realizes a wideband response in the visible range of 400–800 nm but also exceeds most of the reported values of BiI_3_‐based and other iodide‐based photodetectors in key performance parameters such as on/off ratio, responsivity, response speed, and specific detectivity. Our device obtained excellent performance, the reason is that BiI_3_ and CdSe have distinct work functions and Fermi levels before contact, driving significant charge transfer and band bending upon heterojunction formation. This creates a clear Type‐II band alignment. Under illumination, abundant electron–hole pairs are generated. The rising electron density in BiI_3_ lowers the conduction band barrier, whereas increased hole density in CdSe reduces the valence band barrier. As a result, electrons move from BiI_3_ to CdSe, and holes move reversely, enabling efficient interfacial charge transfer and strong photoconductive gain. This significantly enhances the photocurrent, yielding ultrahigh responsivity and quantum efficiency, and thus significantly boosts the performance of the BiI_3_/CdSe heterojunction device. Therefore, this work provides an important reference for the design of new‐generation broadband photodetectors.

**FIGURE 7:**
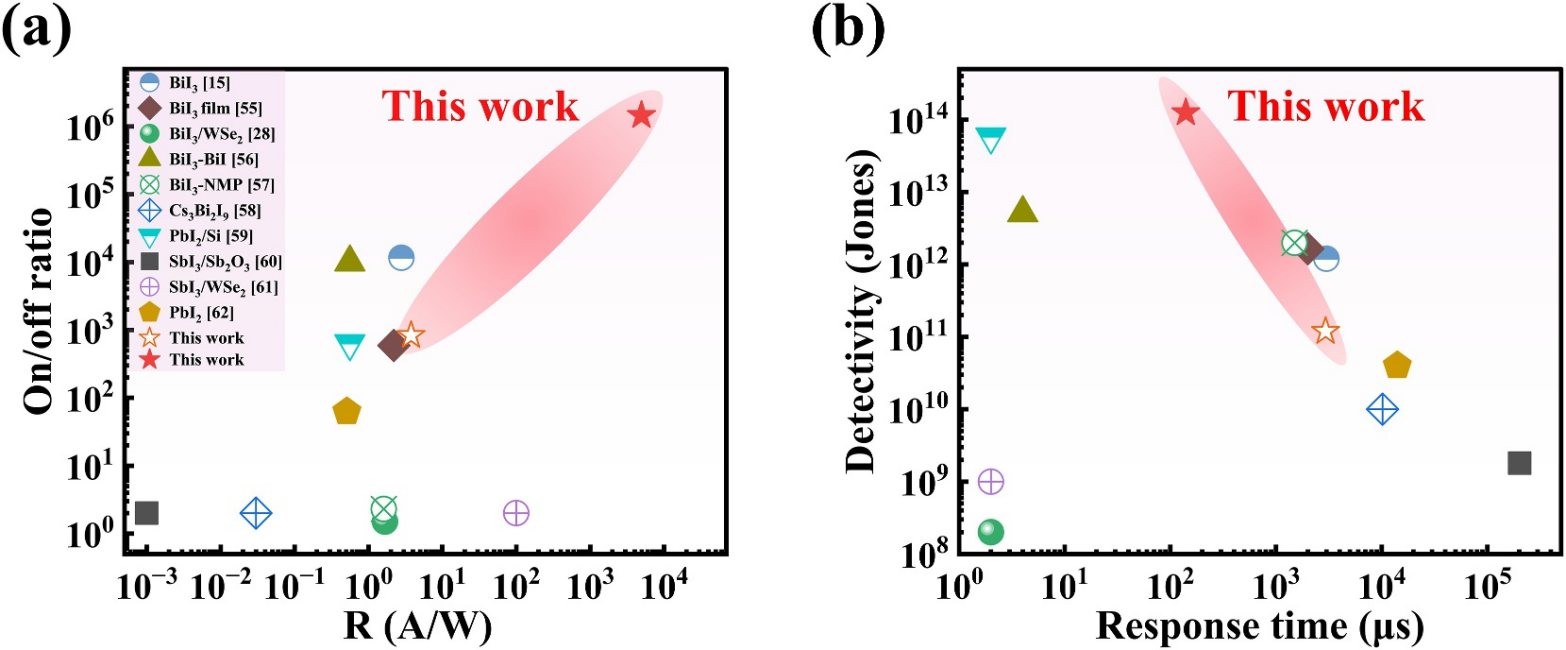
(a, b) Comparison of *R*, on/off ratio, D*, and response time of the BiI_3_/CdSe heterojunction photodetector with other iodide‐based photodetectors.

## 4 Conclusion

In summary, BiI_3_ nanosheets grown perpendicular to the substrate were prepared by physical vapor deposition (PVD), and then a photodetector based on BiI_3_/CdSe heterojunctions was successfully developed by the dry transfer technique. The attained device shows excellent optoelectronic performance at the characteristic wavelengths of 510, 635, and 685 nm. It realizes a wideband detection capability in the visible range of 400–800 nm. Significant breakthroughs have been achieved in key performance metrics, including an ultrahigh responsivity of 4.95 × 10^3^ A/W, a high on/off ratio of 0.97 × 10^6^, a fast response time of 140/200 μs, an external quantum efficiency of 1.45 × 10^6^%, and a specific detectivity of 1.25 × 10^14^ Jones. The figures of merit either meet or surpass the state‐of‐the‐art performance levels of existing BiI_3_‐based photodetectors, which demonstrate remarkable enhancement compared to single‐component devices. Moreover, the photoelectric conversion mechanism revealed by photocurrent scanning imaging and energy band structure provides a deep theoretical explanation for the enhancement of device performance. In addition, the imaging results confirm the excellent imaging performance of the detector with high resolution and high contrast. Therefore, our work establishes the significant potential of BiI_3_/CdSe heterojunction photodetectors as next‐generation high‐performance detectors.

## Author Contributions

**Yifei Huang:** writing – original draft, methodology, investigation, formal analysis. **Haiqiang Huang:** visualization, investigation, data curation. **Lujun Yang:** validation, resources, Investigation. **Lei Liu:** validation, resources. **Hui Zhang:** methodology, resources. **Jie Zheng:** visualization, supervision, investigation. **Yingkai Liu:** writing – review and editing, funding acquisition, supervision.

## Funding

This work was supported by the National Natural Science Foundation of China (Grant Nos. 12264056 and 62164013), the Key Applied Basic Research Program of Science and Technology Commission Foundation of Yunnan Province (No. 202501AS070001), and the Yunnan Expert Workstation Project (202205AF150008).

## Conflicts of Interest

The authors declare no conflicts of interest.

## Supporting information


Supporting Information S1

## Data Availability

The data that support the findings of this study are available from the corresponding author upon reasonable request.
