# Supplementary material for: Ultrahigh‐Performance BiI3/CdSe Heterojunction Photodetectors With Broadband Response
Source: Nanophotonics. 2026 Mar 20;15(6):e70047. doi: 10.1002/nap2.70047 (PMC13642070; doi:10.1002/nap2.70047)
Supplement: Supplementary file 1 [file NAP2-15-e70047-s001.docx]

**Supporting Information**

**Ultrahigh Performance BiI_3_/CdSe Heterojunction Photodetectors with Broadband Response**

Yifei Huang ^1,2,3,4^, Haiqiang Huang ^1,2,3,4^, Lujun Yang ^1,2,4^, Lei Liu ^1,2,3,4^, Hui Zhang ^1,2,3^, Jie Zheng ^1,2,5,*^, Yingkai Liu ^1,2,3,4,**^

^1^ Yunnan Key Laboratory of Opto-electronic Information Technology, Yunnan Normal University, Kunming 650500, China

^2^ Institute of Physics and Electronic Information, Yunnan Normal University, Kunming 650500, China

^3^ Southwest United Graduate School, Kunming 650092, China

^4^ Key Laboratory of Advanced Technique & Preparation for Renewable Energy Materials, Ministry of Education, Yunnan Normal University, Kunming 650500, China

^5^ School Hospital of Yunnan Normal University, Kunming 650500, China.

^*^ Corresponding authors, and ^**^ Corresponding authors.

E-mail: zhengjie197109@163.com; liuyingkai99@163.com, ykliu@ynnu.edu.cn.

Figure S1. Schematic diagram of BiI_3_ nanosheets synthesized by PVD.

Figure S2. Schematic diagram of CdSe nanoribbons prepared by CVD.

Figure S3. Schematic diagram of the fabrication process for the BiI_3_/CdSe device.

Figure S4. (a) Full XPS spectrum of BiI_3_. (b) and (c) XPS spectra of Bi and I. (d) UV-Vis absorption spectra and Tauc plots of CdSe nanoribbons.

Figure S5. (a) CdSe photodetectors in the dark and under 635 nm illumination, respectively. (b) Logarithmic I-V characteristics of photodetectors under different optical power densities. (c) R and EQE of CdSe photodetectors at different optical power densities. (d) D* of CdSe photodetectors at different optical power densities.

Figure S6. (a) Logarithmic I-V curves of the three device groups under 635 nm illumination and in the dark. (b) Current histograms of the three device groups under 635 nm illumination and in the dark.

Figure S7. Comparison of photoelectric performance of BiI_3_ and BiI_3_/CdSe photodetectors under 510 nm irradiation: Log I-V characteristic curves of (a) BiI_3_ and (b) BiI_3_/CdSe photodetectors at different optical power densities, respectively. Experimental and fitted relationships between photocurrent and light intensity for (c) BiI_3_ and (d) BiI_3_/CdSe photodetectors. R and EQE values of (e) BiI_3_ and (f) BiI_3_/CdSe photodetectors at different optical power densities. D* of (g) BiI_3_ and (h) BiI_3_ /CdSe photodetectors at different optical power densities.

Figure S8. Photoelectric properties of BiI_3_/CdSe photodetector under 685 nm irradiation: (a) Log I-V characteristics of BiI_3_/CdSe photodetectors at different optical power densities. (b) Experimental and fitted relations between photocurrent and optical intensity of BiI_3_/CdSe photodetectors. (c) EQE and R of BiI_3_/CdSe photodetectors at different optical power densities. (d) D* of BiI_3_/CdSe photodetectors at different optical power densities.

Figure S9. (a) Photocurrent of BiI_3_ and BiI_3_/CdSe photodetectors under different illumination durations. (b) Photocurrent response of BiI_3_ and BiI_3_/CdSe photodetectors immediately after fabrication and after 30 days of air exposure, measured under 635 nm illumination.

Figure S10. (a) UPS spectrum of BiI_3_. (b) UPS spectrum of CdSe.

Figure S11. Signal-to-noise ratio at different optical power densities.

Figure S12. Measurement conditions for BiI_3_/CdSe heterojunction photodetector and other iodide-based photodetectors.
